# Supplementary material for: Characterization of novel phage Henuyfy11N: a potential therapeutic agent against extended-spectrum β-lactamase (ESBL)-producing Escherichia coli
Source: Microbiol Spectr. 2026 Apr 27;14(6):e03253-25. doi: 10.1128/spectrum.03253-25 (PMC13228064; doi:10.1128/spectrum.03253-25)
Supplement: Tables S1 and S2, and Figure S1 — Tables S1: Strains used for phage Henuyfy11N host range analysis. Tables S2: The open reading frames (ORFs) analysis of phage Henuyfy11N. Fig. S1: Taxonomic prediction of phage Henuyfy11N using taxMyPhage v3.3.6. [file spectrum.03253-25-s0001.docx]

Table S1 Strains used for phage Henuyfy11N host range analysis

| **Strain number** | **Gender of patient** | **Age of patient (year)** | **Source of the strain** | **Species** | **Drug resistance phenotype** | **Susceptibility** | **Origin** |
| --- | --- | --- | --- | --- | --- | --- | --- |
| [2021002N](#药敏谱!A3) | Male | 60 | Midstream urine | *E. coli* | ESBL | - | The First Affiliated Hospital of Henan University |
| 2021006N | Female | 70 | Vaginal discharge | *E. coli* | ESBL | - | The First Affiliated Hospital of Henan University |
| 2021007N | Female | 72 | Midstream urine | *E. coli* | ESBL | - | The First Affiliated Hospital of Henan University |
| 2021008N | Female | 70 | Midstream urine | *E. coli* | ESBL | - | The First Affiliated Hospital of Henan University |
| 2021012N | Female | 73 | Midstream urine | *E. coli* | ESBL | - | The First Affiliated Hospital of Henan University |
| 2021014N | Female | 73 | Midstream urine | *E. coli* | ESBL | - | The First Affiliated Hospital of Henan University |
| 2021016N | Male | 75 | Midstream urine | *E. coli* | ESBL | - | The First Affiliated Hospital of Henan University |
| 2021018N | Female | 77 | Secretions | *E. coli* | ESBL | - | The First Affiliated Hospital of Henan University |
| 2021019N | Female | 74 | Midstream urine | *E. coli* | ESBL | - | The First Affiliated Hospital of Henan University |
| 2021022N | Male | 64 | Midstream urine | *E. coli* | ESBL | - | The First Affiliated Hospital of Henan University |
| 2021023N | Female | 73 | Drainage fluid | *E. coli* | ESBL | - | The First Affiliated Hospital of Henan University |
| 2021024N | Female | 80 | Midstream urine | *E. coli* | ESBL | - | The First Affiliated Hospital of Henan University |
| 2021025N | Male | 78 | Midstream urine | *E. coli* | ESBL | - | The First Affiliated Hospital of Henan University |
| 2021026N | Male | 50 | Blood | *E. coli* | ESBL | - | The First Affiliated Hospital of Henan University |
| 2021027N | Female | 69 | Sputum | *E. coli* | ESBL | - | The First Affiliated Hospital of Henan University |
| 2021028N | Female | 78 | Vaginal discharge | *E. coli* | ESBL | - | The First Affiliated Hospital of Henan University |
| 2021029N | Male | 39 | Pus | *E. coli* | ESBL | - | The First Affiliated Hospital of Henan University |
| 2021030N | Male | 66 | Midstream urine | *E. coli* | ESBL | - | The First Affiliated Hospital of Henan University |
| 2021031N | Male | 73 | Sputum | *E. coli* | ESBL | - | The First Affiliated Hospital of Henan University |
| 2021032N | Female | 69 | Drainage fluid | *E. coli* | ESBL | - | The First Affiliated Hospital of Henan University |
| 2021033N | Female | 88 | Drainage fluid | *E. coli* | ESBL | - | The First Affiliated Hospital of Henan University |
| 2021034N | Male | 23 | Pus | *E. coli* | ESBL | - | The First Affiliated Hospital of Henan University |
| 2021036N | Male | 87 | Sputum | *E. coli* | ESBL | - | The First Affiliated Hospital of Henan University |
| 2021038N | Male | 51 | Pus | *E. coli* | ESBL | - | The First Affiliated Hospital of Henan University |
| 2021040N | Female | 58 | Bile | *E. coli* | ESBL | - | The First Affiliated Hospital of Henan University |
| 2021042N | Male | 73 | Bile | *E. coli* | ESBL | - | The First Affiliated Hospital of Henan University |
| 2021043N | Male | 28 | Sputum | *E. coli* | ESBL | - | The First Affiliated Hospital of Henan University |
| 2021044N | Female | 88 | Bile | *E. coli* | ESBL | - | The First Affiliated Hospital of Henan University |
| 2021046N | Female | 70 | Blood | *E. coli* | ESBL | - | The First Affiliated Hospital of Henan University |
| 2021047N | Male | 68 | Blood | *E. coli* | ESBL | - | The First Affiliated Hospital of Henan University |
| 2021050N | Male | 30 | Pus | *E. coli* | ESBL | - | The First Affiliated Hospital of Henan University |
| 2021051N | Female | 63 | Pus | *E. coli* | ESBL | - | The First Affiliated Hospital of Henan University |
| 2021052N | Male | 70 | Midstream urine | *E. coli* | ESBL | - | The First Affiliated Hospital of Henan University |
| 2021053N | Male | 71 | Midstream urine | *E. coli* | ESBL | - | The First Affiliated Hospital of Henan University |
| 2021054N | Male | 68 | Bile | *E. coli* | ESBL | - | The First Affiliated Hospital of Henan University |
| 2021055N | Male | 67 | Pus | *E. coli* | ESBL | - | The First Affiliated Hospital of Henan University |
| 2021056N | Female | 57 | Midstream urine | *E. coli* | ESBL | - | The First Affiliated Hospital of Henan University |
| 2021057N | Male | 67 | Bile | *E. coli* | ESBL | - | The First Affiliated Hospital of Henan University |
| 2021058N | Male | 88 | Sputum | *E. coli* | ESBL | - | The First Affiliated Hospital of Henan University |
| 2021060N | Female | 71 | Midstream urine | *E. coli* | ESBL | - | The First Affiliated Hospital of Henan University |
| 2021061N | Male | 74 | Sputum | *E. coli* | ESBL | - | The First Affiliated Hospital of Henan University |
| 2021062N | Male | 78 | Midstream urine | *E. coli* | ESBL | - | The First Affiliated Hospital of Henan University |
| 2021064N | Male | 69 | Blood | *E. coli* | ESBL | - | The First Affiliated Hospital of Henan University |
| 2021065N | Male | 60 | Ascites | *E. coli* | ESBL | - | The First Affiliated Hospital of Henan University |
| 2021066N | Male | 35 | Pus | *E. coli* | ESBL | - | The First Affiliated Hospital of Henan University |
| 2021068N | Male | 73 | Midstream urine | *E. coli* | ESBL | - | The First Affiliated Hospital of Henan University |
| 2021069N | Male | 41 | Pus | *E. coli* | ESBL | - | The First Affiliated Hospital of Henan University |
| 2021070N | Male | 84 | Midstream urine | *E. coli* | ESBL | - | The First Affiliated Hospital of Henan University |
| 2021073N | Female | 88 | Sputum | *E. coli* | ESBL | - | The First Affiliated Hospital of Henan University |
| 2021074N | Female | 79 | Midstream urine | *E. coli* | ESBL | - | The First Affiliated Hospital of Henan University |
| 2021075N | Male | 70 | Sputum | *E. coli* | ESBL | - | The First Affiliated Hospital of Henan University |
| 2021076N | Male | 43 | Blood | *E. coli* | ESBL | - | The First Affiliated Hospital of Henan University |
| 2021077N | Male | 88 | Drainage fluid | *E. coli* | ESBL | - | The First Affiliated Hospital of Henan University |
| 2021078N | Female | 66 | Blood | *E. coli* | ESBL | - | The First Affiliated Hospital of Henan University |
| 2021081N | Male | 93 | Blood | *E. coli* | ESBL | - | The First Affiliated Hospital of Henan University |
| 2021082N | Female | 35 | Pus | *E. coli* | ESBL | - | The First Affiliated Hospital of Henan University |
| 2021084N | Female | 69 | Midstream urine | *E. coli* | ESBL | - | The First Affiliated Hospital of Henan University |
| 2021085N | Female | 66 | Sputum | *E. coli* | ESBL | - | The First Affiliated Hospital of Henan University |
| 2021087N | Female | 80 | Drainage fluid | *E. coli* | ESBL | - | The First Affiliated Hospital of Henan University |
| 2021088N | Male | 72 | Ascites | *E. coli* | ESBL | - | The First Affiliated Hospital of Henan University |
| 2021089N | Male | 81 | Pus | *E. coli* | ESBL | - | The First Affiliated Hospital of Henan University |
| 2021090N | Female | 61 | Midstream urine | *E. coli* | ESBL | - | The First Affiliated Hospital of Henan University |
| 2021091N | Female | 34 | Midstream urine | *E. coli* | ESBL | - | The First Affiliated Hospital of Henan University |
| 2021093N | Male | 72 | Puncture fluid | *E. coli* | ESBL | - | The First Affiliated Hospital of Henan University |
| 2021094N | Male | 60 | Bile | *E. coli* | ESBL | - | The First Affiliated Hospital of Henan University |
| 2021096N | Female | 48 | Secretions | *E. coli* | ESBL | - | The First Affiliated Hospital of Henan University |
| 2021097N | Male | 75 | Drainage fluid | *E. coli* | ESBL | - | The First Affiliated Hospital of Henan University |
| 2021099N | Female | 47 | Midstream urine | *E. coli* | ESBL | - | The First Affiliated Hospital of Henan University |
| 2021100N | Male | 77 | Drainage fluid | *E. coli* | ESBL | - | The First Affiliated Hospital of Henan University |
| 2021101N | Female | 29 | Midstream urine | *E. coli* | ESBL | - | The First Affiliated Hospital of Henan University |
| 2021104N | Female | 30 | Midstream urine | *E. coli* | ESBL | - | The First Affiliated Hospital of Henan University |
| 2021105N | Female | 32 | Midstream urine | *E. coli* | ESBL | - | The First Affiliated Hospital of Henan University |
| 2021108N | Male | 70 | Secretions | *E. coli* | ESBL | - | The First Affiliated Hospital of Henan University |
| 2021109N | Male | 55 | Secretions | *E. coli* | ESBL | - | The First Affiliated Hospital of Henan University |
| 2021111N | Male | 66 | Sputum | *E. coli* | ESBL | - | The First Affiliated Hospital of Henan University |
| 2021112N | Male | 41 | Pus | *E. coli* | ESBL | - | The First Affiliated Hospital of Henan University |
| 2021113N | Female | 82 | Secretions | *E. coli* | ESBL | - | The First Affiliated Hospital of Henan University |
| 2021115N | Male | 57 | Drainage fluid | *E. coli* | ESBL | - | The First Affiliated Hospital of Henan University |
| 2021116N | Female | 78 | Midstream urine | *E. coli* | ESBL | - | The First Affiliated Hospital of Henan University |
| 2021117N | Female | 46 | Bile | *E. coli* | ESBL | - | The First Affiliated Hospital of Henan University |
| 2021118N | Female | 51 | Midstream urine | *E. coli* | ESBL | - | The First Affiliated Hospital of Henan University |
| 2021121N | Female | 63 | Bile | *E. coli* | ESBL | - | The First Affiliated Hospital of Henan University |
| 2021122N | Female | 70 | Drainage fluid | *E. coli* | ESBL | - | The First Affiliated Hospital of Henan University |
| 2021124N | Female | 79 | Sputum | *E. coli* | ESBL | - | The First Affiliated Hospital of Henan University |
| 2021125N | Female | 78 | Drainage fluid | *E. coli* | ESBL | - | The First Affiliated Hospital of Henan University |
| 2021126N | Male | 55 | Drainage fluid | *E. coli* | ESBL | - | The First Affiliated Hospital of Henan University |
| 2021128N | Male | 33 | Ascites | *E. coli* | ESBL | - | The First Affiliated Hospital of Henan University |
| 2021130N | Male | 62 | Pus | *E. coli* | ESBL | - | The First Affiliated Hospital of Henan University |
| 2021131N | Female | 78 | Drainage fluid | *E. coli* | ESBL | - | The First Affiliated Hospital of Henan University |
| 2021134N | Female | 79 | Blood | *E. coli* | ESBL | - | The First Affiliated Hospital of Henan University |
| 2021136N | Female | 87 | Sputum | *E. coli* | ESBL | - | The First Affiliated Hospital of Henan University |
| 2021137N | Male | 80 | Blood | *E. coli* | ESBL | - | The First Affiliated Hospital of Henan University |
| 2021138N | Male | 66 | Blood | *E. coli* | ESBL | - | The First Affiliated Hospital of Henan University |
| 2021141N | Male | 63 | Sputum | *E. coli* | ESBL | - | The First Affiliated Hospital of Henan University |
| 2021142N | Female | 76 | Midstream urine | *E. coli* | ESBL | - | The First Affiliated Hospital of Henan University |
| 2021144N | Male | 57 | Secretions | *E. coli* | ESBL | - | The First Affiliated Hospital of Henan University |
| 2021145N | Female | 26 | Midstream urine | *E. coli* | ESBL | - | The First Affiliated Hospital of Henan University |
| 2021146N | Female | 62 | Midstream urine | *E. coli* | ESBL | - | The First Affiliated Hospital of Henan University |
| 2021148N | Male | 32 | Blood | *E. coli* | ESBL | - | The First Affiliated Hospital of Henan University |
| 2021149N | Female | 66 | Bile | *E. coli* | ESBL | - | The First Affiliated Hospital of Henan University |
| 2021150N | Female | 54 | Drainage fluid | *E. coli* | ESBL | - | The First Affiliated Hospital of Henan University |
| 2021151N | Female | 56 | Midstream urine | *E. coli* | ESBL | - | The First Affiliated Hospital of Henan University |
| 2021152N | Male | 50 | Drainage fluid | *E. coli* | ESBL | - | The First Affiliated Hospital of Henan University |
| 2021153N | Male | 57 | Blood | *E. coli* | ESBL | - | The First Affiliated Hospital of Henan University |
| 2021154N | Male | 63 | Midstream urine | *E. coli* | ESBL | - | The First Affiliated Hospital of Henan University |
| 2021157N | Female | 72 | Midstream urine | *E. coli* | ESBL | - | The First Affiliated Hospital of Henan University |
| 2021159N | Female | 34 | Midstream urine | *E. coli* | ESBL | - | The First Affiliated Hospital of Henan University |
| 2021160N | Male | 68 | Blood | *E. coli* | ESBL | - | The First Affiliated Hospital of Henan University |
| 2021163N | Male | 69 | Midstream urine | *E. coli* | ESBL | - | The First Affiliated Hospital of Henan University |
| 2021164N | Male | 68 | Blood | *E. coli* | ESBL | - | The First Affiliated Hospital of Henan University |
| 2021165N | Female | 83 | Vaginal discharge | *E. coli* | ESBL | - | The First Affiliated Hospital of Henan University |
| 2021166N | Female | 86 | Drainage fluid | *E. coli* | ESBL | - | The First Affiliated Hospital of Henan University |
| 2021167N | Male | 76 | Drainage fluid | *E. coli* | ESBL | - | The First Affiliated Hospital of Henan University |
| 2021168N | Female | 50 | Midstream urine | *E. coli* | ESBL | - | The First Affiliated Hospital of Henan University |
| 2021170N | Female | 66 | Sputum | *E. coli* | ESBL | - | The First Affiliated Hospital of Henan University |
| 2021172N | Female | 87 | Sputum | *E. coli* | ESBL | - | The First Affiliated Hospital of Henan University |
| 2021173N | Male | 77 | Midstream urine | *E. coli* | ESBL | - | The First Affiliated Hospital of Henan University |
| 2021175N | Female | 88 | Sputum | *E. coli* | ESBL | - | The First Affiliated Hospital of Henan University |
| 2021176N | Male | 56 | Midstream urine | *E. coli* | ESBL | - | The First Affiliated Hospital of Henan University |
| 2021177N | Female | 73 | Midstream urine | *E. coli* | ESBL | - | The First Affiliated Hospital of Henan University |
| 2021179N | Male | 52 | Sputum | *E. coli* | ESBL | - | The First Affiliated Hospital of Henan University |
| 2021180N | Female | 74 | Midstream urine | *E. coli* | ESBL | - | The First Affiliated Hospital of Henan University |
| 2021181N | Male | 65 | Midstream urine | *E. coli* | ESBL | - | The First Affiliated Hospital of Henan University |
| 2021183N | Male | 45 | Midstream urine | *E. coli* | ESBL | - | The First Affiliated Hospital of Henan University |
| 2021184N | Female | 72 | Blood | *E. coli* | ESBL | - | The First Affiliated Hospital of Henan University |
| 2021186N | Male | 77 | Midstream urine | *E. coli* | ESBL | - | The First Affiliated Hospital of Henan University |
| 2021187N | Male | 93 | Sputum | *E. coli* | ESBL | - | The First Affiliated Hospital of Henan University |
| 2021188N | Male | 80 | Midstream urine | *E. coli* | ESBL | - | The First Affiliated Hospital of Henan University |
| 2021189N | Male | 63 | Drainage fluid | *E. coli* | ESBL | - | The First Affiliated Hospital of Henan University |
| **2021190N** | **Female** | **62** | **Midstream urine** | ***E. coli*** | **ESBL** | **+** | **The First Affiliated Hospital of Henan University** |
| 2021191N | Male | 72 | Sputum | *E. coli* | ESBL | - | The First Affiliated Hospital of Henan University |
| 2021194N | Female | 71 | Blood | *E. coli* | ESBL | - | The First Affiliated Hospital of Henan University |
| 2021195N | Male | 58 | Blood | *E. coli* | ESBL | - | The First Affiliated Hospital of Henan University |
| 2021196N | Male | 58 | Drainage fluid | *E. coli* | ESBL | - | The First Affiliated Hospital of Henan University |
| 2021197N | Male | 64 | Midstream urine | *E. coli* | ESBL | - | The First Affiliated Hospital of Henan University |
| 2021198N | Female | 64 | Midstream urine | *E. coli* | ESBL | - | The First Affiliated Hospital of Henan University |
| 2021199N | Male | 78 | Midstream urine | *E. coli* | ESBL | - | The First Affiliated Hospital of Henan University |
| 2021200N | Female | 79 | Bile | *E. coli* | ESBL | - | The First Affiliated Hospital of Henan University |
| 2024001N | Male | 70 | Sputum | *Acinetobacter baumannii* | NA | - | The First Affiliated Hospital of Henan University |
| 2025001N | Female | 52 | Blood | *E. coli* | NA | - | The First Affiliated Hospital of Henan University |
| 2025002N | Female | 81 | Blood | *E. coli* | ESBL | - | The First Affiliated Hospital of Henan University |
| 2025004N | Female | 92 | Blood | *E. coli* | ESBL | - | The First Affiliated Hospital of Henan University |
| 2025005N | Male | 62 | Sputum | *E. coli* | CRE | - | The First Affiliated Hospital of Henan University |
| 2025008N | Male | 78 | Blood | *E. coli* | NA | - | The First Affiliated Hospital of Henan University |
| 2025009N | Female | 73 | Sputum | *Acinetobacter baumannii* | CRAB | - | The First Affiliated Hospital of Henan University |
| **2025011N** | **Female** | **63** | **Blood** | ***E. coli*** | **ESBL** | **+** | **The First Affiliated Hospital of Henan University** |
| 2025012N | Male | 40 | Midstream urine | *Pseudomonas aeruginosa* | CRPA | - | The First Affiliated Hospital of Henan University |
| 2025014N | Male | 71 | Blood | *E. coli* | ESBL | - | The First Affiliated Hospital of Henan University |
| 2025015N | Male | 76 | Blood | *E. coli* | ESBL | - | The First Affiliated Hospital of Henan University |
| 2025018N | Female | 75 | Blood | *E. coli* | NA | - | The First Affiliated Hospital of Henan University |
| 2025019N | Male | 66 | Sputum | *Pseudomonas aeruginosa* | CRPA | - | The First Affiliated Hospital of Henan University |
| 2025020N | Male | 74 | Sputum | *Pseudomonas aeruginosa* | CRPA | - | The First Affiliated Hospital of Henan University |
| 2025021N | Male | 83 | Sputum | *Acinetobacter baumannii* | CRAB | - | The First Affiliated Hospital of Henan University |
| 2025024N | Female | 73 | Sputum | *E. coli* | CRE | - | The First Affiliated Hospital of Henan University |
| 2025026N | Female | 72 | Secretions | *Acinetobacter baumannii* | CRAB | - | The First Affiliated Hospital of Henan University |
| 2025029N | Male | 91 | Sputum | *Acinetobacter baumannii* | CRAB | - | The First Affiliated Hospital of Henan University |
| 2025031N | Female | 81 | Midstream urine | *E. coli* | ESBL | - | The First Affiliated Hospital of Henan University |
| 2025032N | Female | 54 | Midstream urine | *E. coli* | ESBL | - | The First Affiliated Hospital of Henan University |
| 2025035N | Male | 64 | Drainage fluid | *Acinetobacter baumannii* | NA | - | The First Affiliated Hospital of Henan University |
| 2025044N | Female | 72 | Sputum | *Acinetobacter baumannii* | CRAB | - | The First Affiliated Hospital of Henan University |
| 2025046N | Male | 68 | Sputum | *Acinetobacter baumannii* | NA | - | The First Affiliated Hospital of Henan University |
| 2025050N | Male | 48 | Secretions | *Acinetobacter baumannii* | CRAB | - | The First Affiliated Hospital of Henan University |
| 2025054N | Male | 76 | BALF | *Acinetobacter baumannii* | CRAB | - | The First Affiliated Hospital of Henan University |
| 2025065N | Female | 59 | Sputum | *Acinetobacter baumannii* | CRAB | - | The First Affiliated Hospital of Henan University |
| 2025066N | Male | 77 | Sputum | *Acinetobacter baumannii* | CRAB | - | The First Affiliated Hospital of Henan University |
| 2025074N | Male | 64 | Sputum | *Acinetobacter baumannii* | NA | - | The First Affiliated Hospital of Henan University |

NA: Not available; ESBL: Extended-spectrum β-lactamases; CRAB: Carbapenem-resistant *Acinetobacter baumannii*; CRPA: Carbapenem-resistant *Pseudomonas aeruginosa*; CRE: Carbapenem-resistant *Enterobacteriaceae*

Table S2 The open reading frames (ORFs) analysis of phage Henuyfy11N

| ORF | Start | End | Protein  (kDa) | Annotated function | Representative similarity to  protein sin database | Query  cover | Percent Identity | Accession  no. |
| --- | --- | --- | --- | --- | --- | --- | --- | --- |
| 1 | 1 | 387 | 13.987 | spanin | *Escherichia* phage BUCT789 | 100.00% | 100.00% | XQU49878.1 |
| 2 | 576 | 1202 | 22.331 | head scaffolding protein | *Escherichia* phage K1H | 100.00% | 100.00% | [YP_009168840.1](https://www.ncbi.nlm.nih.gov/protein/YP_009168840.1?report=genbank&log$=prottop&blast_rank=4&RID=2X2XCSD5013) |
| 3 | 1209 | 2177 | 33.862 | tail protein | *Escherichia* phage BUCT789 | 100.00% | 100.00% | [XQU49880.1](https://www.ncbi.nlm.nih.gov/protein/XQU49880.1?report=genbank&log$=prottop&blast_rank=1&RID=2X328HW4016) |
| 4 | 2405 | 2917 | 17.689 | head-tail adaptor Ad1 | *Escherichia* phage BUCT789 | 100.00% | 100.00% | [XQU49882.1](https://www.ncbi.nlm.nih.gov/protein/XQU49882.1?report=genbank&log$=prottop&blast_rank=1&RID=2X3NGD14016) |
| 5 | 2920 | 3534 | 20.870 | prohead core protein protease | *Escherichia* phage BUCT789 | 100.00% | 99.51% | [XQU49883.1](https://www.ncbi.nlm.nih.gov/protein/XQU49883.1?report=genbank&log$=prottop&blast_rank=1&RID=2X3XBYJ6013) |
| 6 | 3480 | 3893 | 15.029 | head-to-tail connector complex protein | *Escherichia* phage phiWAO78-1 | 100.00% | 88.32% | [QQV88041.1](https://www.ncbi.nlm.nih.gov/protein/QQV88041.1?report=genbank&log$=prottop&blast_rank=2&RID=2X4JG6FR013) |
| 7 | 3890 | 4285 | 14.686 | [tail component](https://blast.ncbi.nlm.nih.gov/Blast.cgi#alnHdr_UTQ77400) | [*Escherichia* phage hz69](https://www.ncbi.nlm.nih.gov/Taxonomy/Browser/wwwtax.cgi?id=2951240) | 100.00% | 100.00% | [UTQ77400.1](https://www.ncbi.nlm.nih.gov/protein/UTQ77400.1?report=genbank&log$=prottop&blast_rank=1&RID=2X5H7MY1016) |
| 8 | 4282 | 4698 | 15.098 | [tail protein](https://blast.ncbi.nlm.nih.gov/Blast.cgi#alnHdr_YP_010749203) | *Escherichia* phage NTEC3 | 99.00% | 100.00% | [YP_010749203.1](https://www.ncbi.nlm.nih.gov/protein/YP_010749203.1?report=genbank&log$=prottop&blast_rank=5&RID=2X64UJ5W016) |
| 9 | 4701 | 5867 | 40.743 | [MAG: tail tube protein](https://blast.ncbi.nlm.nih.gov/Blast.cgi#alnHdr_UVM98886) | Bacteriophage sp | 100.00% | 100.00% | [UVM98886.1](https://www.ncbi.nlm.nih.gov/protein/UVM98886.1?report=genbank&log$=prottop&blast_rank=1&RID=2X6D1FX7016) |
| 10 | 4940 | 5392 | 15.701 | [hypothetical protein](https://blast.ncbi.nlm.nih.gov/Blast.cgi#alnHdr_XDJ01654) | [*Salmonella* phage vB_SE130_2P](https://www.ncbi.nlm.nih.gov/Taxonomy/Browser/wwwtax.cgi?id=3236707) | 29.00% | 63.64% | [XDJ01654.1](https://www.ncbi.nlm.nih.gov/protein/XDJ01654.1?report=genbank&log$=prottop&blast_rank=1&RID=2X74AAN9013) |
| 11 | 6061 | 6291 | 8.687 | [MAG: protein of unknown function DUF3310](https://blast.ncbi.nlm.nih.gov/Blast.cgi#alnHdr_UVN10507) | Bacteriophage sp. | 100.00% | 98.68% | [UVN10507.1](https://www.ncbi.nlm.nih.gov/protein/UVN10507.1?report=genbank&log$=prottop&blast_rank=2&RID=2X7RWNFU016) |
| 12 | 6288 | 7418 | 42.400 | [calcineurin-like phosphoesterase](https://blast.ncbi.nlm.nih.gov/Blast.cgi#alnHdr_XQU49890) | [*Escherichia* phage BUCT789](https://blast.ncbi.nlm.nih.gov/Blast.cgi#alnHdr_XQU49890) | 100.00% | 100.00% | [XQU49890.1](https://www.ncbi.nlm.nih.gov/protein/XQU49890.1?report=genbank&log$=prottop&blast_rank=1&RID=2X8AWYS4013) |
| 13 | 7484 | 7810 | 11.571 | [glycoprotein precursor](https://blast.ncbi.nlm.nih.gov/Blast.cgi#alnHdr_XPO97471) | [*Shigella* phage phiSD1](https://blast.ncbi.nlm.nih.gov/Blast.cgi#alnHdr_XPO97471) | 100.00% | 97.22% | [XPO97471.1](https://www.ncbi.nlm.nih.gov/protein/XPO97471.1?report=genbank&log$=prottop&blast_rank=8&RID=2X8T1AKR013) |
| 14 | 7975 | 8391 | 15.564 | tail assembly chaperone | [*Escherichia* phage K1H](https://www.ncbi.nlm.nih.gov/Taxonomy/Browser/wwwtax.cgi?id=698487) | 100.00% | 100.00% | [YP_009168854.1](https://www.ncbi.nlm.nih.gov/protein/YP_009168854.1?report=genbank&log$=prottop&blast_rank=1&RID=2XBT0A84016) |
| 15 | 8490 | 8753 | 9.792 | [tail assembly chaperone](https://blast.ncbi.nlm.nih.gov/Blast.cgi#alnHdr_XPK40469) | *Escherichia* phage AnnaReinhart_Bas75 | 100.00% | 100.00% | [XPK40469.1](https://www.ncbi.nlm.nih.gov/protein/XPK40469.1?report=genbank&log$=prottop&blast_rank=3&RID=2XD9BY4M016) |
| 16 | 8746 | 11055 | 82.296 | [tail length tape measure protein](https://blast.ncbi.nlm.nih.gov/Blast.cgi#alnHdr_UTQ77390) | *Escherichia* phage hz69 | 100.00% | 100.00% | [UTQ77390.1](https://www.ncbi.nlm.nih.gov/protein/UTQ77390.1?report=genbank&log$=prottop&blast_rank=1&RID=2XDXGANN016) |
| 17 | 9450 | 10319 | 30.888 | [hypothetical protein](https://blast.ncbi.nlm.nih.gov/Blast.cgi#alnHdr_XRA40029) | *Salmonella* phage PK2 | 58.00% | 73.37% | [XRA40029.1](https://www.ncbi.nlm.nih.gov/protein/XRA40029.1?report=genbank&log$=prottop&blast_rank=1&RID=2XEJXWJ7016) |
| 18 | 11059 | 12450 | 50.259 | [minor tail protein](https://blast.ncbi.nlm.nih.gov/Blast.cgi#alnHdr_XQU49895) | *Escherichia* phage BUCT789 | 100.00% | 100.00% | [XQU49895.1](https://www.ncbi.nlm.nih.gov/protein/XQU49895.1?report=genbank&log$=prottop&blast_rank=1&RID=2XF1JXJ4016) |
| 19 | 12454 | 12969 | 18.821 | [minor tail protein](https://blast.ncbi.nlm.nih.gov/Blast.cgi#alnHdr_XPO96475) | *Escherichia* phage EF2-1 | 100.00% | 95.32% | [XPO96475.1](https://www.ncbi.nlm.nih.gov/protein/XPO96475.1?report=genbank&log$=prottop&blast_rank=6&RID=2XFJM3ME016) |
| 20 | 12966 | 13331 | 14.327 | [Gamma-D-glutamyl-L-lysine dipeptidyl-peptidase](https://blast.ncbi.nlm.nih.gov/Blast.cgi#alnHdr_XPO96673) | *Escherichia* phage EM1 | 100.00% | 98.35% | [XPO96673.1](https://www.ncbi.nlm.nih.gov/protein/XPO96673.1?report=genbank&log$=prottop&blast_rank=3&RID=2XFZ0ZD2016) |
| 21 | 13394 | 15874 | 90.667 | [tail fiber protein](https://blast.ncbi.nlm.nih.gov/Blast.cgi#alnHdr_XQU49898) | *Escherichia* phage BUCT789 | 100.00% | 99.27% | [XQU49898.1](https://www.ncbi.nlm.nih.gov/protein/XQU49898.1?report=genbank&log$=prottop&blast_rank=1&RID=2XG7BU3C016) |
| 22 | 15887 | 17686 | 64.886 | [tailspike protein](https://blast.ncbi.nlm.nih.gov/Blast.cgi#alnHdr_XQU49899) | *Escherichia* phage BUCT789 | 100.00% | 99.00% | XQU49899.1 |
| 23 | 17849 | 18364 | 19.478 | [DNA primase](https://blast.ncbi.nlm.nih.gov/Blast.cgi#alnHdr_WFD55389) | *Escherichia* phage JSSK01 | 100.00% | 98.83% | [WFD55389.1](https://www.ncbi.nlm.nih.gov/protein/WFD55389.1?report=genbank&log$=prottop&blast_rank=1&RID=2XGSVN0D016) |
| 24 | 18361 | 19785 | 53.390 | [DNA helicase](https://blast.ncbi.nlm.nih.gov/Blast.cgi#alnHdr_WQN07569) | [*Escherichia* phage vB-Eco-KMB36](https://www.ncbi.nlm.nih.gov/Taxonomy/Browser/wwwtax.cgi?id=3093646) | 100.00% | 98.52% | [WQN07569.1](https://www.ncbi.nlm.nih.gov/protein/WQN07569.1?report=genbank&log$=prottop&blast_rank=14&RID=2Y2GZXHD016) |
| 25 | 20013 | 20267 | 9.22 | [hypothetical protein QCF74_gp43](https://blast.ncbi.nlm.nih.gov/Blast.cgi#alnHdr_YP_010749248) | [*Escherichia* phage SZH-1](https://www.ncbi.nlm.nih.gov/Taxonomy/Browser/wwwtax.cgi?id=2945916) | 100.00% | 95.24% | [YP_010749248.1](https://www.ncbi.nlm.nih.gov/protein/YP_010749248.1?report=genbank&log$=prottop&blast_rank=4&RID=2Y32ZJ3Y013) |
| 26 | 20269 | 20583 | 12.009 | VRR-NUC domain protein | [*Escherichia* phage hz69](https://www.ncbi.nlm.nih.gov/Taxonomy/Browser/wwwtax.cgi?id=2951240) | 100.00% | 100.00% | [UTQ77379.1](https://www.ncbi.nlm.nih.gov/protein/UTQ77379.1?report=genbank&log$=prottop&blast_rank=1&RID=2Y448VZM016) |
| 27 | 20698 | 20943 | 9.073 | NA | NA | NA | NA | NA |
| 28 | 20936 | 23209 | 85.723 | [DNA polymerase I](https://blast.ncbi.nlm.nih.gov/Blast.cgi#alnHdr_WPK33117) | [*Escherichia* phage AV105](https://www.ncbi.nlm.nih.gov/Taxonomy/Browser/wwwtax.cgi?id=3077250) | 100.00% | 99.74% | [WPK33117.2](https://www.ncbi.nlm.nih.gov/protein/WPK33117.1?report=genbank&log$=prottop&blast_rank=2&RID=2Y4MJJF3013) |
| 29 | 23268 | 23897 | 23.643 | [ssDNA binding and annealing protein](https://blast.ncbi.nlm.nih.gov/Blast.cgi#alnHdr_XQU49909) | *Escherichia* phage BUCT789 | 100.00% | 100.00% | [XQU49909.1](https://www.ncbi.nlm.nih.gov/protein/XQU49909.1?report=genbank&log$=prottop&blast_rank=1&RID=2Y5JZ6FR013) |
| 30 | 23988 | 25229 | 45.883 | [nuclease superfamily protein](https://blast.ncbi.nlm.nih.gov/Blast.cgi#alnHdr_UTQ77373) | *Escherichia* phage hz69 | 100.00% | 97.09% | [UTQ77373.1](https://www.ncbi.nlm.nih.gov/protein/UTQ77373.1?report=genbank&log$=prottop&blast_rank=3&RID=2Y62VKZZ013) |
| 31 | 25226 | 25498 | 10.738 | [MAG TPA: hypothetical protein](https://blast.ncbi.nlm.nih.gov/Blast.cgi#alnHdr_DAH69327) | Bacteriophage sp. | 100.00% | 98.89% | [DAH69327.1](https://www.ncbi.nlm.nih.gov/protein/DAH69327.1?report=genbank&log$=prottop&blast_rank=1&RID=2Y7U45VH016) |
| 32 | 25542 | 26060 | 19.389 | [13.88 kDa late protein](https://blast.ncbi.nlm.nih.gov/Blast.cgi#alnHdr_WKW35194) | *Escherichia* phage CR01 | 100.00% | 93.60% | [WKW35194.1](https://www.ncbi.nlm.nih.gov/protein/WKW35194.1?report=genbank&log$=prottop&blast_rank=7&RID=2Y8275VE013) |
| 33 | 26185 | 26400 | 7.759 | [helix-turn-helix domain-containing protein](https://blast.ncbi.nlm.nih.gov/Blast.cgi#alnHdr_UTQ77368) | *Escherichia* phage hz69 | 100.00% | 98.59% | [UTQ77368.1](https://www.ncbi.nlm.nih.gov/protein/UTQ77368.1?report=genbank&log$=prottop&blast_rank=2&RID=2Y8CVCXG013) |
| 34 | 26418 | 28676 | 83.388 | [putative helicase-primase](https://blast.ncbi.nlm.nih.gov/Blast.cgi#alnHdr_UTQ77367) | *Escherichia* phage hz69 | 100.00% | 99.73% | [UTQ77367.1](https://www.ncbi.nlm.nih.gov/protein/UTQ77367.1?report=genbank&log$=prottop&blast_rank=1&RID=30M4F8D6016) |
| 35 | 28735 | 28968 | 8.503 | putative uvsX-like protein | *Escherichia* phage EM1 | 100.00% | 98.70% | [XPO96696.1](https://www.ncbi.nlm.nih.gov/protein/XPO96696.1?report=genbank&log$=prottop&blast_rank=2&RID=30P8BZK9013) |
| 36 | 29824 | 30078 | 9.442 | [MAG: hypothetical protein](https://blast.ncbi.nlm.nih.gov/Blast.cgi#alnHdr_UVX86706) | Bacteriophage sp. | 100.00% | 100.00% | [UVX86706.1](https://www.ncbi.nlm.nih.gov/protein/UVX86706.1?report=genbank&log$=prottop&blast_rank=1&RID=30PT9R1Z013) |
| 37 | 30127 | 30360 | 8.411 | [Alpa family transcriptional regulator](https://blast.ncbi.nlm.nih.gov/Blast.cgi#alnHdr_XQU49854) | *Escherichia* phage BUCT789 | 100.00% | 100.00% | [XQU49854.1](https://www.ncbi.nlm.nih.gov/protein/XQU49854.1?report=genbank&log$=prottop&blast_rank=1&RID=30R0MUCM013) |
| 38 | 30944 | 31219 | 10.345 | [lactocepin](https://blast.ncbi.nlm.nih.gov/Blast.cgi#alnHdr_AQN31798) | *Escherichia* phage G_AB-2017 | 100.00% | 88.89% | [AQN31798.1](https://www.ncbi.nlm.nih.gov/protein/AQN31798.1?report=genbank&log$=prottop&blast_rank=8&RID=30SB4AHK016) |
| 39 | 31360 | 31719 | 13.784 | [cell division protein](https://blast.ncbi.nlm.nih.gov/Blast.cgi#alnHdr_UTQ77431) | *Escherichia* phage hz69 | 100.00% | 74.38% | [UTQ77431.1](https://www.ncbi.nlm.nih.gov/protein/UTQ77431.1?report=genbank&log$=prottop&blast_rank=4&RID=30S041PN016) |
| 40 | 31701 | 31919 | 8.011 | [hypothetical protein](https://blast.ncbi.nlm.nih.gov/Blast.cgi#alnHdr_WPK33137) | *Escherichia* phage AV104 | 93.00% | 100.00% | [WPK33137.1](https://www.ncbi.nlm.nih.gov/protein/WPK33137.1?report=genbank&log$=prottop&blast_rank=10&RID=30STDG8G013) |
| 41 | 31922 | 32176 | 9.740 | [hypothetical protein HSE2_gp020](https://blast.ncbi.nlm.nih.gov/Blast.cgi#alnHdr_AUE23490) | *Escherichia* phage vB_EcoS_HSE2 | 100.00% | 100.00% | [AUE23490.1](https://www.ncbi.nlm.nih.gov/protein/AUE23490.1?report=genbank&log$=prottop&blast_rank=1&RID=30T9GWBV013) |
| 42 | 32176 | 32457 | 10.751 | [hypothetical protein HSE2_gp022](https://blast.ncbi.nlm.nih.gov/Blast.cgi#alnHdr_AUE23492) | *Escherichia* phage vB_EcoS_HSE2 | 100.00% | 100.00% | [AUE23492.1](https://www.ncbi.nlm.nih.gov/protein/AUE23492.1?report=genbank&log$=prottop&blast_rank=1&RID=30TJJAS2016) |
| 43 | 32834 | 33106 | 9.964 | [class I holin-like protein](https://blast.ncbi.nlm.nih.gov/Blast.cgi#alnHdr_YP_010749429) | *Escherichia* phage ZCEC5 | 100.00% | 100.00% | [YP_010749429.1](https://www.ncbi.nlm.nih.gov/protein/YP_010749429.1?report=genbank&log$=prottop&blast_rank=1&RID=30TVSG3T013) |
| 44 | 33069 | 33569 | 17.579 | endolysin | *Escherichia* phage BUCT789 | 100.00% | 100.00% | [XQU49863.1](https://www.ncbi.nlm.nih.gov/protein/XQU49863.1?report=genbank&log$=prottop&blast_rank=1&RID=30URZF7X013) |
| 45 | 34214 | 34450 | 9.016 | [Nin protein](https://blast.ncbi.nlm.nih.gov/Blast.cgi#alnHdr_YP_010749701) | *Escherichia* phage vB_EcoS_XY1 | 74.00% | 94.83% | [YP_010749701.1](https://www.ncbi.nlm.nih.gov/protein/YP_010749701.1?report=genbank&log$=prottop&blast_rank=10&RID=30UY0FR1013) |
| 46 | 34450 | 34689 | 8.800 | [hypothetical protein](https://blast.ncbi.nlm.nih.gov/Blast.cgi#alnHdr_WPK33146) | *Escherichia* phage AV104 | 100.00% | 100.00% | [WPK33146.1](https://www.ncbi.nlm.nih.gov/protein/WPK33146.1?report=genbank&log$=prottop&blast_rank=1&RID=30VHZCDR016) |
| 47 | 34625 | 34858 | 8.709 | [hypothetical protein HSE2_gp027](https://blast.ncbi.nlm.nih.gov/Blast.cgi#alnHdr_AUE23497) | *Escherichia* phage vB_EcoS_HSE2 | 100.00% | 89.61% | AUE23497.1 |
| 48 | 34863 | 35144 | 10.562 | hypothetical protein | *Escherichia* phage BUCT789 | 100.00% | 100.00% | [XQU49869.1](https://www.ncbi.nlm.nih.gov/protein/XQU49869.1?report=genbank&log$=prottop&blast_rank=1&RID=30W43PFF016) |
| 49 | 35374 | 35670 | 11.16 | [zinc finger protein](https://blast.ncbi.nlm.nih.gov/Blast.cgi#alnHdr_XQU49871) | *Escherichia* phage BUCT789 | 100.00% | 100.00% | [XQU49871.1](https://www.ncbi.nlm.nih.gov/protein/XQU49871.1?report=genbank&log$=prottop&blast_rank=1&RID=30Y9WKRU013) |
| 50 | 35875 | 36435 | 20.599 | [terminase](https://blast.ncbi.nlm.nih.gov/Blast.cgi#alnHdr_YP_009597281) | *Escherichia* phage K1ind1 | 100.00% | 97.31% | [YP_009597281.1](https://www.ncbi.nlm.nih.gov/protein/YP_009597281.1?report=genbank&log$=prottop&blast_rank=1&RID=30YEWBX4013) |
| 51 | 36432 | 37682 | 45.893 | [putative terminase](https://blast.ncbi.nlm.nih.gov/Blast.cgi#alnHdr_YP_010749450) | *Escherichia* phage vB_EcoS-Ro145c2YLVW | 100.00% | 99.76% | [YP_010749450.1](https://www.ncbi.nlm.nih.gov/protein/YP_010749450.1?report=genbank&log$=prottop&blast_rank=2&RID=30ZDW1E3016) |
| 52 | 37694 | 39187 | 54.902 | [DUF4055 domain-containing protein](https://blast.ncbi.nlm.nih.gov/Blast.cgi#alnHdr_XQU49875) | *Escherichia* phage BUCT789 | 100.00% | 100.00% | [XQU49875.1](https://www.ncbi.nlm.nih.gov/protein/XQU49875.1?report=genbank&log$=prottop&blast_rank=1&RID=30ZSBXVB013) |
| 53 | 39224 | 40300 | 39.638 | minor capsid protein | *Escherichia* phage BUCT789 | 97.00% | 100.00% | [XQU49876.1](https://www.ncbi.nlm.nih.gov/protein/XQU49876.1?report=genbank&log$=prottop&blast_rank=4&RID=3100YHAF016) |
| 54 | 40300 | 40761 | 16.286 | [tail protein](https://blast.ncbi.nlm.nih.gov/Blast.cgi#alnHdr_XQU49877) | *Escherichia* phage BUCT789 | 100.00% | 100.00% | [XQU49877.1](https://www.ncbi.nlm.nih.gov/protein/XQU49877.1?report=genbank&log$=prottop&blast_rank=1&RID=310DBMWY016) |

NA: Not available


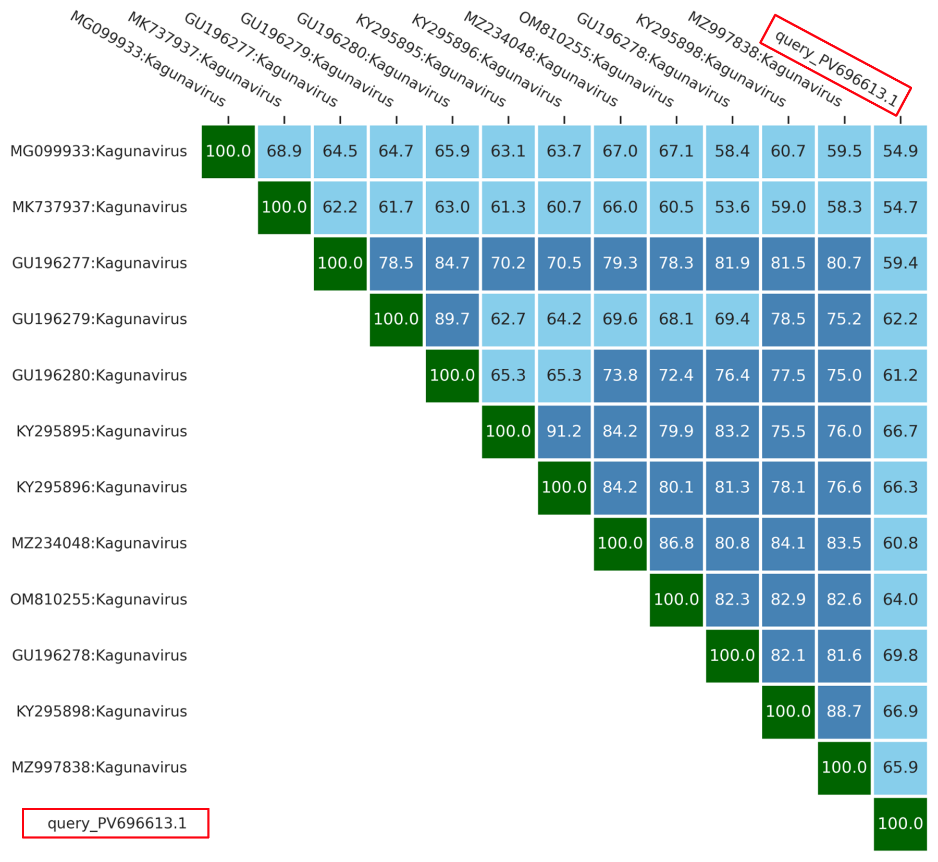


**Figure S1 Taxonomic prediction of phage Henuyfy11N using taxMyPhage v3.3.6.** The heatmap presents the pairwise nucleotide sequence similarity matrix between the phage Henuyfy11N and reference phage genomes in the database. Color intensity corresponds to the percentage of nucleotide sequence identity (see color scale). Each row and column represents a distinct reference phage genome (or taxonomic unit). Prediction confidence is determined according to the International Committee on Taxonomy of Viruses (ICTV) demarcation criteria for phage genera (70% nucleotide identity) and species (95% nucleotide identity).
